# Ceftriaxone to PRevent pneumOnia and inflammaTion aftEr Cardiac arresT (PROTECT): study protocol for a randomized, placebo-controlled trial

**DOI:** 10.1186/s13063-022-06127-w

**Published:** 2022-03-04

**Authors:** David J. Gagnon, Sergey V. Ryzhov, Meghan A. May, Richard R. Riker, Bram Geller, Teresa L. May, Sarah Bockian, Joanne T. deKay, Ashley Eldridge, Thomas Van der Kloot, Patricia Lerwick, Christine Lord, F. Lee Lucas, Patrick Mailloux, Barbara McCrum, Meghan Searight, Joel Wirth, Jonathan Zuckerman, Douglas Sawyer, David B. Seder

**Affiliations:** 1 Department of Pharmacy, Maine Medical Center, Portland, ME USA; 2 Maine Medical Center Research Institute, Scarborough, ME USA; 3 Tufts University School of Medicine, Boston, MA USA; 4 University of New England College of Osteopathic Medicine, Biddeford, ME USA; 5 Department of Critical Care Services, Maine Medical Center, Portland, ME USA; 6 Maine Medical Partners, MaineHealth Cardiology, Scarborough, ME USA; 7 Maine Medical Center Neuroscience Institute, Maine Medical Center, Portland, ME USA

**Keywords:** Cardiac arrest, Pneumonia, Targeted temperature management, Hypothermia, Antibiotics, Inflammation, Microbiome, Metagenomics, Ceftriaxone

## Abstract

**Background:**

Pneumonia is the most common infection after out-of-hospital cardiac arrest (OHCA) occurring in up to 65% of patients who remain comatose after return of spontaneous circulation. Preventing infection after OHCA may (1) reduce exposure to broad-spectrum antibiotics, (2) prevent hemodynamic derangements due to local and systemic inflammation, and (3) prevent infection-associated morbidity and mortality.

**Methods:**

The ceftriaxone to PRevent pneumOnia and inflammaTion aftEr Cardiac arrest (PROTECT) trial is a randomized, placebo-controlled, single-center, quadruple-blind (patient, treatment team, research team, outcome assessors), non-commercial, superiority trial to be conducted at Maine Medical Center in Portland, Maine, USA. Ceftriaxone 2 g intravenously every 12 h for 3 days will be compared with matching placebo. The primary efficacy outcome is incidence of early-onset pneumonia occurring < 4 days after mechanical ventilation initiation. Concurrently, T cell-mediated inflammation bacterial resistomes will be examined. Safety outcomes include incidence of type-one immediate-type hypersensitivity reactions, gallbladder injury, and C*lostridioides difficile*-associated diarrhea. The trial will enroll 120 subjects over approximately 3 to 4 years.

**Discussion:**

The PROTECT trial is novel in its (1) inclusion of OHCA survivors regardless of initial heart rhythm, (2) use of a low-risk antibiotic available in the USA that has not previously been tested after OHCA, (3) inclusion of anti-inflammatory effects of ceftriaxone as a novel mechanism for improved clinical outcomes, and (4) complete metagenomic assessment of bacterial resistomes pre- and post-ceftriaxone prophylaxis. The long-term goal is to develop a definitive phase III trial powered for mortality or functional outcome.

**Trial registration:**

ClinicalTrials.gov NCT04999592. Registered on August 10, 2021.

## Administrative information

Note: the numbers in curly brackets in this protocol refer to SPIRIT checklist item numbers. The order of the items has been modified to group similar items (see http://www.equator-network.org/reporting-guidelines/spirit-2013-statement-defining-standard-protocol-items-for-clinical-trials/).

**Table:** 

Title {1}	ceftriaxone to PRevent pneumOnia and inflammaTion aftEr Cardiac arresT (PROTECT): a randomized-controlled trial and microbiome assessment
Trial registration {2a and 2b}.	ClinicalTrials.gov (NCT04999592) registered August 10, 2021
Protocol version {3}	This manuscript describes the PROTECT trial protocol version 3 dated 6/11/21.
Funding {4}	The trial is supported by the National Institute of General Medical Sciences (1P20GM139745-01).
Author details {5a}	David J. Gagnon • Department of Pharmacy, Maine Medical Center, Portland, ME, USA • Maine Medical Center Research Institute, Scarborough, ME, USA • Tufts University School of Medicine, Boston, MA, USA Sergey V. Ryzhov • Maine Medical Center Research Institute, Scarborough, ME, USA Meghan A. May • University of New England College of Osteopathic Medicine, Biddeford, ME, USA Richard R. Riker • Tufts University School of Medicine, Boston, MA, USA • Department of Critical Care Services, Maine Medical Center, Portland, ME, USA Bram Geller • Tufts University School of Medicine, Boston, MA, USA • Maine Medical Partners, MaineHealth Cardiology, Scarborough, ME, USA Teresa L. May • Maine Medical Center Research Institute, Scarborough, ME, USA • Tufts University School of Medicine, Boston, MA, USA • Department of Critical Care Services, Maine Medical Center, Portland, ME, USA Sarah Bockian • Maine Medical Center Neuroscience Institute, Maine Medical Center, Portland, ME, USA Joanne T. deKay • Maine Medical Center Research Institute, Scarborough, ME, USA Ashley Eldridge • Maine Medical Center Neuroscience Institute, Maine Medical Center, Portland, ME, USA Thomas Van der Kloot • Department of Critical Care Services, Maine Medical Center, Portland, ME, USA Patricia Lerwick • Department of Critical Care Services, Maine Medical Center, Portland, ME, USA Christine Lord • Maine Medical Center Neuroscience Institute, Maine Medical Center, Portland, ME, USA F. Lee Lucas • Maine Medical Center Research Institute, Scarborough, ME, USA Patrick Mailloux • Department of Critical Care Services, Maine Medical Center, Portland, ME, USA Barbara McCrum • Maine Medical Center Neuroscience Institute, Maine Medical Center, Portland, ME, USA Meghan Searight • Maine Medical Center Neuroscience Institute, Maine Medical Center, Portland, ME, USA Joel Wirth • Department of Critical Care Services, Maine Medical Center, Portland, ME, USA Jonathan Zuckerman • Department of Critical Care Services, Maine Medical Center, Portland, ME, USA Douglas Sawyer • Maine Medical Center Research Institute, Scarborough, ME, USA • Maine Medical Partners, MaineHealth Cardiology, Scarborough, ME, USA David B. Seder • Maine Medical Center Research Institute, Scarborough, ME, USA • Tufts University School of Medicine, Boston, MA, USA • Department of Critical Care Services, Maine Medical Center, Portland, ME, USA
Name and contact information for the trial sponsor {5b}	National Institute of General Medical Sciences 45 Center Drive MSC 6200 Bethesda, MD 20892-6200 Phone: 301-496-7301 Email: info@nigms.nih.gov
Role of sponsor {5c}	The funding agency was not involved with study design, data collection, analysis and interpretation of data, writing of the manuscript, or the decision to submit the manuscript for publication.

## Background and rationale {6a}

Pneumonia results in alveolar inflammation and fluid or purulent material accumulation in the lungs [1]. It is the most common infection after cardiac arrest, occurring in up to 65% of patients treated with targeted temperature management (TTM) [2]. Infections are associated with increased intensive care unit (ICU) length of stay (LOS), hospital LOS, duration of mechanical ventilation, post-discharge rehabilitation need, tracheostomy need, and mortality, while also reducing the incidence of a good functional outcome [3–7].

Pneumonia likely results from aspiration during cardiopulmonary resuscitation (CPR) or by introduction of oropharyngeal flora into the lungs during airway management. It may also be due to gastrointestinal hypoperfusion, which leads to ischemic injury to the intestinal mucosa, bacterial translocation, and hematogenous spread of bacteria [8–13]. Infection might also be the result of post-resuscitation immune suppression, but this requires confirmation [14].

Preventing early pneumonia may (1) reduce exposure to broad-spectrum antibiotics and subsequent collateral damage, (2) prevent hemodynamic derangements due to local and systemic inflammation, and (3) prevent an association between infection and morbidity and mortality. These benefits must be carefully balanced with the risk for altering bacterial resistomes, genetic material producing resistance, in the absence of clinical infection.

## Objectives {7}

The primary objective is to determine if prophylactic ceftriaxone administered within 6 h of ICU admission reduces the incidence of early-onset pneumonia (EOP). The secondary objectives are to quantify T cell-mediated inflammation and bacterial resistomes as assessed by resistance genotypes in stool and sputum.

## Trial design {8}

The ceftriaxone to PRevent pneumOnia and inflammaTion aftEr Cardiac arresT (PROTECT) is a randomized, placebo-controlled, single-center, quadruple-blind (patient, treatment team, research team, outcome assessors), non-commercial, superiority trial.

## Study setting {9}

The trial will be conducted at Maine Medical Center in Portland, Maine, USA. The hospital has 637 licensed beds including a 12-bed cardiac ICU and 32-bed mixed medical, surgical, and neurological ICU. It is affiliated with Tufts University School of Medicine in Boston, Massachusetts, USA. Maine Medical Center has managed over 1000 patients with TTM after OHCA and it is the largest hospital in Northern New England. All OHCA patients are admitted to or co-managed by the Neurocritical Care service. The Neurocritical Care team has around-the-clock coverage by an attending physician and an advanced practice provider (i.e., Physician Assistant or Nurse Practitioner).

## Eligibility criteria {10}

Inclusion criteria:

- ≥18 years of age
- Comatose (do not follow simple verbal commands)
- Have any initial heart rhythm (shockable or non-shockable)
- Out-of-hospital cardiac arrest (OHCA) including the emergency department

Exclusion criteria:

- Name on the Exception from Informed Consent (EFIC) opt-out list
- In-hospital cardiac arrest
- Interval > 6 h from ICU admission to study drug initiation
- Preexisting terminal disease making 180-day survival unlikely
- Legally authorized representative (LAR) refused informed consent
- Emergent coronary artery bypass grafting
- Anaphylaxis or angioedema to beta-lactam antibiotics (i.e., cephalosporins or penicillins)
  - Beta-lactam allergies listed without a known reaction will not be an exclusion
- Under legal guardianship or prisoner
- Known colonization with methicillin-resistant *Staphylococcus aureus* (MRSA)
- Clinical bacterial infection prior to hospital admission defined as any one of the following:
  - Infectious prodrome preceding OHCA
  - Active course of antibiotics for infection prior to admission
  - Active infection documented in the electronic medical record
  - Family or surrogate endorsement of an active infection
- Active course of antibiotics for infectious or non-infectious indications
- Clinical indication for antibiotics at the time of screening in the opinion of the treatment team

### Inclusion window

Controlled studies in heterogeneous cohorts of acutely brain-injured patients used four or 6 h from intubation or 6 h from ICU admission as the prophylaxis window [15–17]. The Antibiotherapy during Therapeutic Hypothermia to Prevent Infectious Complications (ANTHARTIC) trial, which included only OHCA patients with shockable rhythms, used < 6 h from return of spontaneous circulation (ROSC) to randomization [18]. Consistent with prior studies, patients will be enrolled in the PROTECT trial if ≤6 h has passed from time of ICU admission to study drug initiation.

### Who will take informed consent? {26a}

Study investigators will approach the patient’s Power of Attorney for Health Care (POAHC) or Legally Authorized Representative (LAR) to provide informed consent. Patients regaining consciousness will be asked for informed consent as soon as they have the capacity to do so. On the consent form, subjects will be asked if they agree to use of their data should they choose to withdraw from the trial. Subjects will also be asked for permission for the research team to share relevant data with people from the center, institute, and university taking part in the research, or from regulatory authorities, where relevant.

In the event a patient’s POAHC or LAR cannot be reached for informed consent within 30 min, the EFIC process will be initiated per the Food and Drug Administration Guidance for Institutional Review Boards, Clinical Investigators, and Sponsors on Exception from Informed Consent Requirements for Emergency Research [19]. As soon as a POAHC or LAR is found they will be informed of the trial and consented. After community consultation and public disclosure, the Institutional Review Board (IRB) at Maine Medical Center deemed it was appropriate to conduct the trial.

### Additional consent provisions for collection and use of participant data and biological specimens {26b}

This trial involves collecting biological blood and sputum samples. In the informed consent form, participants are asked whether they agree to the use of their data and biological specimens for future studies.

## Interventions

### Explanation for the choice of comparators {6b}

Ceftriaxone was selected for many reasons: (1) bactericidal activity against commonly isolated bacteria in comatose OHCA patients, (2) generic availability and low cost, (3) ease of administration over 30 min, (4) favorable local susceptibility profile, (5) excellent safety data, and (6) potential neuro-protective effects [20]. Data for bacterial distribution in TTM-1, ANTHARTIC, and local susceptibility to ceftriaxone appear in Table 1 [18, 21]. Bacterial distribution was not reported in the original TTM-2 trial publication [22].

**Table 1:** Bacteria identified in the TTM-1 and ANTHARTIC trials with local susceptibility data to ceftriaxone

Bacteria	TTM-1 trial^**a**^	ANTHARTIC trial^**a**^	MMC ceftriaxone susceptibility^**b**^
**Gram-positive**			
*Staphylococcus aureus*	22.9%	12%	86%
*Streptococcus pneumoniae*	5.5%	7%	99%
*Streptococcus agalactiae*	1.5%	3%	Not reported
**Gram-negative**			
*Haemophilus influenzae*	9.1%	22%	100%
*Escherichia coli*	9.1%	11%	93%
*Klebsiella pneumoniae*	5.1%	4%	94%
*Serratia marcescens*	5.1%	3%	89%
*Klebsiella oxytoca*	3.6%	1%	97%
*Enterobacter cloacae*	3.3%	3%	78%
*Pseudomonas aeruginosa*	2.5%	3%	Not active
*Enterobacter aerogenes*	2.2%	2%	75%
*Proteus mirabilis*	2.2%	1%	98%
*Moraxella catarrhalis*	1.5%	1%	Not reported

^a^Percentages do not equal 100 for each study as some organisms (e.g., fungi) were not included above

^b^Maine Medical Center ceftriaxone antibiogram data through December 2019

*MMC* Maine Medical Center, *TTM* targeted temperature management

### Intervention description {11a}

**Fig. 1:**
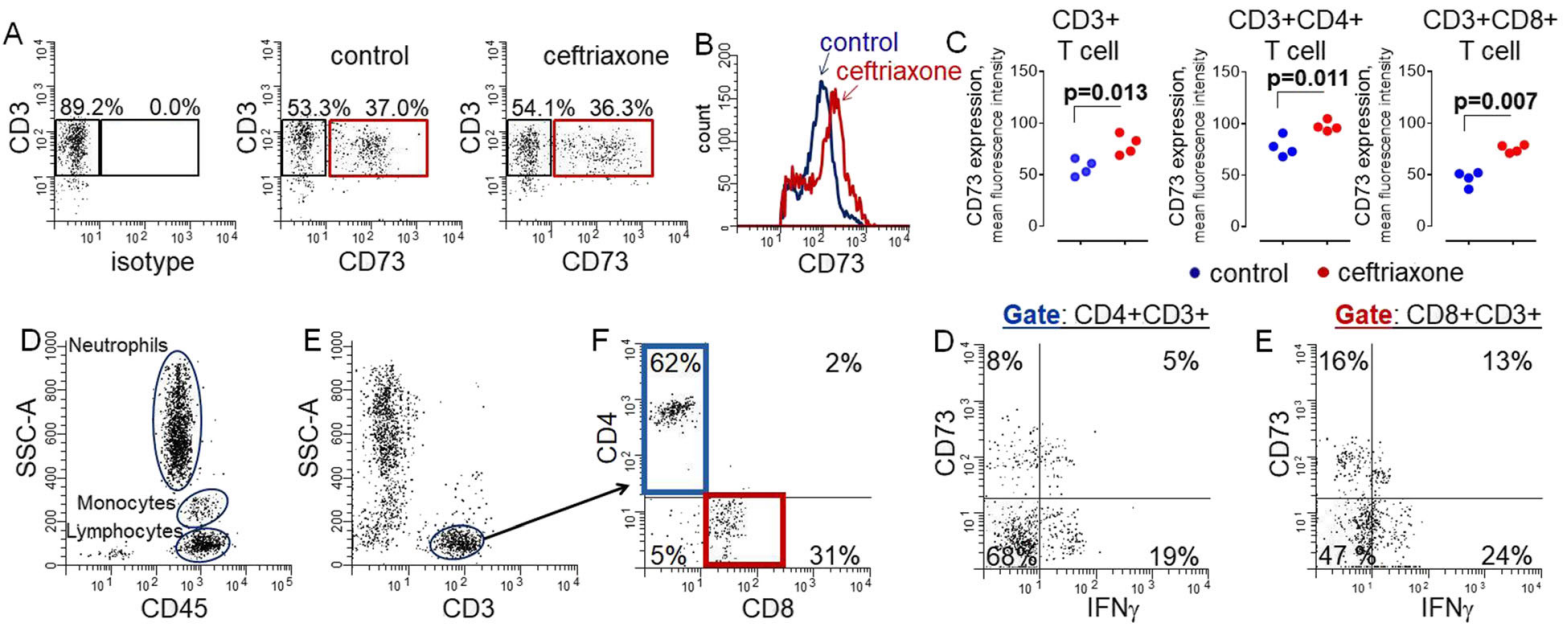
Ceftriaxone increases CD73 on T lymphocytes, and CD73 levels correlate with IFN-γ. **A** T cells were purified from peripheral blood from pre-operative coronary artery bypass patients and incubated in the absence (control) or presence of 50 μg/ml ceftriaxone for 24 h. **B** CD73 was measured in viable CD3 T cells gated as shown (red gate). **C** Quantification of flow cytometric data showing CD73 in total CD3 T cells and subpopulations of CD4+ and CD8+ unpaired *t* test, *n*=4. D Subpopulations of peripheral blood cells. **E** CD3+ T lymphocytes were gated and subpopulations of CD4+ (blue gate) and CD8+ (red gate) identified (**F**). **G**, **H** The expression of CD73 and production of IFN-γ in subpopulations of CD4+ (blue gate) and CD8+ (red gate) T lymphocytes

Ceftriaxone for injection is a sterile, semi-synthetic, broad-spectrum cephalosporin antibiotic available for intravenous (IV) or intramuscular injection [20]. The ceftriaxone dose of 2 g IV every 12 h was selected using internal antibiogram data at Maine Medical Center, the cumulative fraction of response based on dose for methicillin-sensitive *Staphylococcus aureus*, and preliminary data suggesting it has anti-inflammatory properties (Fig. 1 and Table 2).

**Table 2:** Cumulative fraction of response to ceftriaxone at various doses for methicillin-sensitive *Staphylococcus aureus*

Regimen	Cumulative fraction of response
Ceftriaxone 1 g IV q24h	8%
Ceftriaxone 2 g IV q24h	45%
Ceftriaxone 2 g IV q12h	86%

Matching placebo of 50 mL 0.9% sodium chloride will be dispensed for patients randomized to the control arm. In order to maintain blinding, an opaque cover will be placed over the bag due to ceftriaxone’s light yellow to amber color.

### Criteria for discontinuing or modifying allocated interventions {11b}

Study drug will be discontinued if:

- Gallbladder toxicity occurs OR;
- Withdrawal from the trial is in the subject’s best interest OR;
- Infection develops warranting a change in the antibiotic regimen OR;
- Subject, POAHC, or LAR declines or revokes informed consent.

In the event of stopping the study drug, patients will continue to be followed for safety and efficacy outcomes, but will not have blood, sputum culture, or rectal samples for the inflammation and microbiome assessments.

### Strategies to improve adherence to interventions {11c}

A clinical research associate appointed by Maine Medical Center’s Institutional Review Board (IRB) may visit during the study to ensure proper conduct. Items for review may include:

- Informed consent forms and EFIC documentation
- Compliance with the study protocol and procedures
- Quality of data collected in the case report form
  - Accuracy
  - Missing data
  - Consistency of the data with the source documents
- Management of the study drug
- Each visit will be recorded in a written monitoring report

### Relevant concomitant care permitted or prohibited during the trial {11d}

Patients will receive early coronary catheterization, when appropriate, and will undergo multimodal delayed neurologic prognostication according to clinical practice guidelines. All care will be the same in each study group except for the study drug.

Patients unable to follow simple verbal commands after ROSC will be treated with TTM. Patients will be sedated and may receive a dose of a paralytic or chilled saline (4 °C) and cooled to a target temperature per the bedside treatment team (33 °C to 37.5 °C). The Bard Medical Arctic Sun Temperature Management System®, a servo-controlled surface cooling device, will be used.

Propofol is preferred and is titrated by bedside nurses. Analgesia is provided with intermittent injections of fentanyl or a continuous infusion. As needed doses of midazolam may be administered. Low doses are maintained to avoid accumulation in the setting of end-organ dysfunction and temperature-mediated decreases in drug metabolism and elimination.

Patients may be monitored with cEEG until they have awoken or it is deemed not clinically necessary. Tracings are read by epileptologists throughout the day and concerning findings are reported to the bedside treatment team. Seizures and epileptiform activity will be classified according to the International League Against Epilepsy, Neurocritical Care Society, and American Clinical Neurophysiology Society’s criteria. Antiepiletpic medications may be administered.

Clinical practice guidelines suggest keeping the MAP > 65 mmHg, at a minimum, with observational data supporting a MAP 80–90 mmHg. MAP will be maintained > 80 mmHg, unless it compromises the hemodynamics. Vasoactive medications, inotropes or mechanical circulatory support may be needed.

Patients who remain comatose will have their neurological prognosis assessed per the ERC’s and the European Society for Intensive Care Medicine recommendations. The multimodal neurological prognostication algorithm will be activated > 72 h after ROSC to avoid confounding from acute metabolic disturbances, sedation, analgesia and paralysis.

### Provisions for post-trial care {30}

Patients will be followed for the duration of their hospitalization and up to 6 months after hospital discharge. At the time of hospital discharge, a research coordinator will determine each subject’s modified Rankin scale (mRS) score and cerebral performance category (CPC). This will also occur at 6 months after hospital discharge via telephone call. In the case of injury or illness resulting from this research study, the subject’s insurance company will be responsible for any costs resulting from underlying disease or treatments provided outside of this research study.

### Outcomes {12}

#### Primary efficacy outcome

- Clinically-diagnosed EOP occurring < 4 days after initiation of mechanical ventilation

There are no validated diagnostic criteria for pneumonia in comatose OHCA survivors or those receiving some form of temperature modulation. Consequently, the definition of clinical pneumonia was adapted from diagnostic criteria for nosocomial pneumonia proposed by the Centers for Disease Control and Prevention/ National Healthcare Safety Network, American Thoracic Society and the Infectious Diseases Society of America, and published definitions of pneumonia in this patient population [18,23,24].

During the 72-h TTM period, clinical pneumonia will be defined as:

New or progressive lung infiltrate and *at least one of the following* (a or b):

- New purulent secretions or change in quantity or quality of sputum
- Worsening gas exchange defined as (any of i through iii):
  i. Oxygen desaturations or PaO_2_/FIO_2_ ≤240
  ii. Increased oxygen requirements
    1. Increase in daily minimum FiO2 of ≥0.20 (20 points) over the daily minimum FiO_2_ of the first day in the baseline period, sustained for ≥ 2 calendar days. Daily minimum defined by lowest value of FiO_2_ during a calendar day that is maintained for > 1 h.
  iii. Increased mechanical ventilator demand
    1. A sustained increase in the daily minimum PEEP of ≥ 3 cmH_2_O following a period of stability or improvement on the ventilator. PEEP values from 0 to 5 cmH2O are considered equivalent. Daily minimum defined by lowest value of PEEP during a calendar day that is maintained for > 1 h.

After the 72-h TTM period, clinical pneumonia will be defined as:

New or progressive lung infiltrate and *at least two of the following* (a through e):

- New purulent secretions or change in quantity or quality of sputum
- Worsening gas exchange, not otherwise explained, defined as (any of i through iii):
  i. Oxygen desaturations or PaO_2_ /FIO_2_ ≤240
  ii. Increased oxygen requirements
  iii. Increased ventilator demand
- Cough, dyspnea, tachypnea, rales, or bronchial breath sounds
- Body temperature > 38 °C
- Leukopenia (< 4000 white blood cells/mm^3^) or leukocytosis (> 12,000 white blood cells/mm^3^)

#### Secondary outcomes

A clinical diagnosis of pneumonia will be microbiologically confirmed using flexible fiberoptic bronchoscopy with > 10^4^ colony forming units (CFU) per mL of pathogenic bacteria or unprotected mini-bronchoalveolar lavage with > 10^4^ CFU/mL of pathogenic bacteria in intubated patients [23, 24]. In non-intubated patients, an expectorated sputum sample will be used. Blinded, board certified pulmonologists will adjudicate any clinical or microbiological pneumonia diagnosis.

- Microbiologically-confirmed EOP occurring < 4 days after initiation of mechanical ventilation
- Microbiologically-confirmed late-onset pneumonia occurring ≥4 days after initiation of mechanical ventilation
- Clinically-diagnosed late-onset pneumonia occurring ≥4 days after initiation of mechanical ventilation
- Incidence of non-pulmonary infections
- ICU-free days in the first 28 days of admission
- Mechanical ventilator-free days in the first 28 days of admission
- ICU LOS
- Hospital LOS
- ICU mortality
- Hospital mortality
- Discharge disposition
- Functional outcome at hospital discharge and 6 months after hospital discharge
  - Good functional outcome will mRS ≤ 0–3 of or a CPC 1–2
  - Functional outcome will be assessed by a research coordinator at hospital discharge and via telephone at 6 months

#### Safety outcomes

- *Clostridioides difficile*-associated diarrhea [25]
  - Diagnosed according to the 2017 Infectious Diseases Society of American Clinical Practice Guidelines for *Clostridium difficile* Infection in Adults:
    - Unexplained and new-onset ≥3 unformed stools in 24 h
    - Positive *Clostridioides difficile* toxin gene polymerase chain reaction assay
- Type one (immediate-type) hypersensitivity reactions [26]
  - Acute symptom onset (minutes to hours) involving the skin, mucosal tissue, or both (e.g., generalized hives, pruritus or flushing, swollen lips-tongue-uvula) and at least one of the following (a through c):
    - Respiratory compromise
    - Reduced blood pressure
    - Signs of end-organ dysfunction
- Gallbladder disease [20]
  - Diagnosed sonographically as an echo without acoustical shadowing suggesting sludge and the presence of ceftriaxone-calcium salt

### Participant timeline {13}

The sequence of events can be found in Table 3.

**Table 3:** Sequence of events in the PROTECT trial

	By 6 h of ICU admission	Day 1 (0–24 h)	Day 2 (24–48 h)	Day 3 (48–72 h)	Day 4 (72–96 h)	Day 7 (168 h)	Day 1 to hospital discharge	Hospital discharge	6-month follow-up
Screening/enrollment	X								
Study drug		X	X	X					
Sputum culture	X				X				
Rectal swab	X				X	X			
Blood sample	X	X		X					
EOP		X	X	X					
Non-pulm. infection							X		
ICU LOS							X		
Ventilator-free days							X		
Hospital LOS							X		
ICU mortality							X		
Hospital mortality							X		
Discharge disposition							X		
CDAD							X		
Type-one allergy		X	X	X					
Gallbladder disease							X		
mRS								X	X
CPC								X	X

*CDAD Clostridioides difficile*-associated diarrhea, *EOP* early-onset pneumonia, *ICU* intensive care unit, *LOS* length of stay, *mRS* modified Rankin scale, *CPC* cerebral performance category

### Sample size {14}

In the ANTHARTIC trial, the incidence of EOP at 5 days was 17% in the prophylaxis group and 31% in controls (absolute difference of 14%) [18]. In contrast to our protocol, when using their definition of EOP, the incidence at 7 days was 19% in the prophylaxis group and 34% in controls (absolute difference of 15%) [18].

In the TTM-1 trial, pneumonia occurred in 52% of patients treated at 33 °C and 46% in those treated at 36 °C [21]. Pneumonia was not defined as early- or late-onset. In a post hoc analysis, the incidence of pneumonia in patients admitted to centers administering antibiotic prophylaxis was 41% compared to 54% at centers that did not [27]. Clustering within centers was possible.

Using data from the International Cardiac Arrest Registry (INTCAR), the incidence of pneumonia, which was not defined as early- or late-onset, was 33% with antibiotic prophylaxis [28]. Accordingly, 60 patients will be randomized to each group (*n*=120 total). This provides 80% power at a two-tailed 5% level of significance to detect an absolute reduction in the incidence of EOP by 25%, from 55 to 30%.

### Recruitment {15}

The study team is notified via electronic mail when a patient has orders for temperature management following OHCA. Additionally, the study team is alerted by a medical communications technician when a patient is being transported by emergency medical services to the hospital after an OHCA. In the event of slow recruitment, a second enrolling center may be activated following IRB approval.

## Assignment of interventions: allocation

### Sequence generation {16a}

A computer-generated allocation sequence will randomize patients in a 1:1 ratio in blocks of six. Randomization will not be stratified.

### Concealment mechanism {16b}

The clinical trials pharmacist will be responsible for randomizing patients independent of study investigators. The clinical trials pharmacist will prepare the study drug for delivery to the bedside nurse for administration.

### Implementation {16c}

A random order sequence was developed by the lead statistician. Patients will be enrolled by research team members, including physicians and pharmacists, as delineated in the Delegation of Authority log. The clinical trials pharmacist with dispense study drug according to the allocation sequence.

## Assignment of interventions: blinding

### Who will be blinded? {17a}

The PROTECT trial is quadruple-blind including the patient/LAR, bedside treatment team, research team, and outcome assessors (i.e., pneumonia adjudicators and functional outcome assessor). Study drug will be dispensed with an amber cover to mask the yellow tint to reconstituted and diluted ceftriaxone.

### Procedure for unblinding if needed {17b}

Unblinding may occur for a serious adverse event or if the bedside treatment team determines it is medically necessary. Results of unblinding will not be communicated to study investigators, members of the pneumonia adjudication committee, or the outcome evaluators. Accidental unblinding will be dealt with on a case-by-case basis and patients may continue in the study depending on the circumstance.

## Data collection and management

### Plans for assessment and collection of outcomes {18a}

Data collection forms will be completed for patients who are randomized. Data will be transcribed into REDCap, which is a secure, web-based application for data capture. Screening logs will be maintained and reasons for exclusion will be recorded. Data collected may follow the International Liaison Committee on Resuscitation’s Utstein criteria for OHCA. Data will be collected by clinical research coordinators or study investigators from medical records, family members, ambulance run reports, or other sources.

### Plans to Promote Participant Retention and Complete Follow-up {18b}

Patients will be followed for the duration of their hospitalization and up to 6 months after hospital discharge. At the time of hospital discharge, a research coordinator will determine each patient’s mRS score and CPC. This will also occur at 6 months after hospital discharge via telephone call.

### Data management {19}

Data will be transcribed into REDCap, which is a secure, web-based application for data capture. Screening logs will also be maintained in REDCap and reasons for exclusion will be recorded. Upon data exportation, a random PROTECT trial identification number will be assigned. Data entered into the REDCap database will include checks for value ranges and empty fields.

### Confidentiality {27}

Documents will be retained at Maine Medical Center for 15 years, which is in compliance with the United States’ Food and Drug Administration Code of Federal Regulations (21 CFR §312.62[c]). Access to source documents may be permitted for trial-related monitoring and audits, when appropriate. Individual data for monitoring, carrying out quality control, and auditing biomedical research may be shared at the discretion of the study investigators.

### Plans for collection, laboratory evaluation, and storage of biological specimens for genetic or molecular analysis in this trial/future use {33}

#### Microbiome and resistome assessment

Sputum and rectal swabs will be collected prior to study drug initiation, within 24 h of completing study drug, and on study-day seven or at the time of withdrawal of life sustaining therapies, whichever occurs first (rectal swab). Total nucleic acids will be extracted and resistomes determined by shotgun metagenomic sequencing using super high-throughput methods.

Total nucleic acids will be extracted from sputum and from rectal swabs using ZymoBIOMICS reagents (Zymo), and shotgun libraries will be prepared and include inline barcodes. Libraries will be pooled in groups of 96 for super high-throughput sequencing with paired-end reads (150 bp) using the Illumina HiSeq 4000 platform.

Library preparation and sequencing reads will be performed at the UC Davis Genome Center. Reads will be de-multiplexed by barcode and trimmed by ILLUMACLIP, and contaminating human sequence reads will be filtered. Remaining reads will be assembled into metagenomes using Velvet, and resistance genotypes will be identified and quantified using ResFams and ShortBRED, respectively [29–32].

#### Inflammation assessment

Peripheral blood samples (10 mL) will be obtained before study drug, and on study-day 1 and study-day 3 (30 mL total) after discontinuation of study drug. Sub-populations of white blood cells will be measured using flow cytometry. Mononuclear cells will be isolated using Ficoll-Paque™. Generation of adenosine by T lymphocyte will be measured using Malachite green phosphate assay (Sigma) [33]. Analysis of genes involved in T cell response will be performed with total RNA isolated from CD3+ lymphocytes using RNeasy Mini Kit (Qiagen). Gene expression will be analyzed using Oligo GEArray® Human T-cell and B-cell Activation Microarray (SABiosciences, OHS-053).

Multi-parametric flow cytometric analysis will be conducted on whole blood cells before the study drug and on study-day 1 and study-day 3. Cells will be stained using fluorochrome-conjugated antibodies. For intracellular staining, cells will be permeabilized using BD Cytofix/Cytoperm™ buffer. The percentage of lymphocyte subsets will be determined within viable cell populations using the MACSQuant 10 analyzer. Myeloid cells (neutrophils and monocytes) will be analyzed to validate ceftriaxone’s effect in lymphocytes.

## Statistical methods

### Statistical methods for primary and secondary outcomes {20a}

The primary efficacy endpoint (pneumonia within 4 days) will be analyzed on an intention-to-treat basis. We will use survival analysis techniques to evaluate the primary endpoint, with failure defined as incident pneumonia and failure time defined as the day on which pneumonia is first identified. Although follow-up time is short, survival analysis will allow us to account for the competing risk of death. Patients who die within 4 days will be censored at the day of death. Patients who do not die and do not develop pneumonia within 4 days will be censored at day 4. We will use standard survival analysis techniques to visually examine differences in pneumonia incidence and will estimate the hazard ratio as our primary outcome. Two-tailed tests of significance will be used, and *p*≤0.05 will be considered significant. Analyses will be performed using SAS version 9.4 and R version 4.0.2.

The secondary efficacy endpoints will be analyzed on an intention-to-treat basis. Late-onset pneumonia and other infectious outcomes will be analyzed using the same statistical approach as the primary efficacy outcome. Mortality at day 28 will be analyzed using the chi-square test.

### Interim analyses {21b}

A single interim analysis will be conducted when 60 (50%) subjects have been enrolled. The independent Data Safety Monitoring Board will be empowered to stop the trial under the following circumstances:

- Harm
  - There is a statistically significant increased risk for serious adverse events, including *Clostridium difficile*-associated diarrhea, gallbladder toxicity, or type one hypersensitivity reactions, during the interim analysis of the first *n*=60 patients enrolled. The *p* value threshold for harm will be set at 0.05.
- Safety
  - Significant safety concerns emerge and the Data Safety Monitoring Board (DSMB) and IRB choose to pause or stop the trial.

The trial will not be terminated for given the intermediate outcome of interest is pneumonia, rather than death or other concrete outcomes (e.g., stroke, myocardial infarction). Additionally, it will not allow for a complete assessment of the microbiome or inflammation.

### Methods for additional analyses (e.g., subgroup analyses) {20b}

Abundance of antibiotic resistance-associated genotypes in resistomes will be compared within and between treatment groups. If ceftriaxone or placebo alters resistomes, ANOVA will be performed across genotypes. The abundance of each genotype will be compared between ceftriaxone- and placebo-treated patients post-intervention by both Student’s *T* test and *χ*^2^ to establish direct changes associated with ceftriaxone and whether those deviate from potential changes in resistomes due to ICU admission. All statistical analyses will be performed using GraphPad Prism 8 (GraphPad Software Inc).

We expect to obtain 56.3 ± 4.5 × 10^6^ of total white blood cells, containing 14.7 ± 1.7 × 10^6^ mononuclear cells and 6.3 ± 1.1 × 10^6^ of CD3+ T cells from 10 mL of blood. We will use approximately 1 × 10^6^ CD3+ T cells to determine direct effects of ceftriaxone on CD73 expression and adenosine generation. We will use approximately 3 × 10^6^ of CD3+ T cells to isolate mRNA for gene expression analysis after treatment with ceftriaxone. Approximately 2–3 × 10^6^ whole blood cells will be used for flow cytometric analysis. Statistical analysis will be performed using the GraphPad Prism 7.0 software (GraphPad Software Inc). Comparisons between groups will be performed using two-tailed unpaired *t* tests. Comparisons between several groups will be performed using one-way ANOVA followed by appropriate post hoc tests.

### Methods in analysis to handle protocol non-adherence and any statistical methods to handle missing data {20c}

Missing data will be reported at the time of publication. If further statistical analyses reveal substantial missing data, multiple imputation will be considered.

### Plans to give access to the full protocol, participant-level data, and statistical code {31c}

The full trial protocol will be published as a supplement along with the results of the primary and secondary analyses in a single publication. Participant-level data will be made available according to the National Institutes of Health’s Policy for Data Management and Sharing. Data will be made accessible as soon as possible, and no later than the time of an associated publication, or the end of the trial, whichever comes first.

## Oversight and monitoring

### Composition of the coordinating center and trial steering committee {5d}

Maine Medical Center is located in Portland, Maine, USA and has 637 licensed beds including a 12-bed cardiac ICU and 32-bed mixed medical, surgical, and neurological ICU. Its catchment area includes the state of Maine and northeast New Hampshire including approximately 1.3 million people. The Neurocritical Care team has around-the-clock coverage by an attending physician and an advanced practice provider (i.e., Physician Assistant or Nurse Practitioner).

The inflammation analysis will be completed at Maine Medical Center Research Institute in Scarborough, Maine, USA. The microbiome analyses will be done at the University of New England, Biddeford, Maine, USA, with library preparation and sequencing reads performed at the UC Davis Genome Center in Davis, CA, USA.

A Clinical Trial Steering Committee consisting of the principal investigator and co-investigators will oversee the trial. Weekly Neurocritical Care research meetings will be used to discuss enrollment and safety issues. A pneumonia adjudication board will review each diagnosis of pneumonia in a blinded manner. A statistician and data analyst from the Maine Medical Center Research Institute will analyze the data in a blinded manner for the interim analysis and final analysis.

### Composition of the data monitoring committee, its role and reporting structure {21a}

A independent DSMB will execute a Data Safety Monitoring Plan (DSMP) and be chaired by a physician. The DSMB will consist of a board certified infectious diseases physician, board certified pulmonologist, and a senior biostatistician. The DSMB will meet at least every 4 months and more frequently during the interim analysis. More information on the DSMB and DSMP can be found in the trial protocol.

### Adverse event reporting and harms {22}

Reportable serious adverse events will be reported to the IRB and DSMB within five business days of the principal investigator notification. Reportable serious adverse events resulting in death will be reported to the IRB and DSMB within 48 h of the principal investigator notification. Unexpected serious adverse events deemed reasonably or definitely associated with the study drug will be reported to the IRB and DSMB within five business days of the principal investigator notification. Approximately 60% of patients who survive to the hospital after out-of-hospital cardiac arrest will survive. Death (other than as noted above) will be a serious adverse event and reported to the IRB and DSMB within five business days.

### Frequency and plans for auditing trial conduct {23}

As described in the “Strategies to Improve Adherence to Interventions {11c}” section, a clinical research associate appointed by Maine Medical Center’s Institutional Review Board (IRB) may visit during the study to ensure proper conduct. Additionally, the DSMB will be empowered to share information related to study conduct with the Clinical Trial Steering Committee and the IRB. Information shared should focus on trial procedures, and may include the following:

- Rates of recruitment, ineligibility, noncompliance, protocol violations and dropouts
- Completeness and timeliness of data
- Degree of concordance between site evaluation of events and centralized review
- Balance between study arms on important prognostic variables
- Accrual within important subsets

### Plans for communicating important protocol amendments to relevant parties {25}

Protocol amendments will be approved by the Maine Medical Center IRB. Protocol training will occur after any protocol amendment. The funder, National Institute of General Medical Sciences, will be notified of the first and last enrollment. The ClinicalTrials.gov listing will be updated periodically for enrollment or major protocols changes.

### Dissemination plans {31a}

Analyses will be performed 6 months after hospital discharge of the last patient. The full-length manuscript will be submitted to a peer-reviewed international medical journal. Authorship will follow the guidelines set forth by the International Committee of Medical Journal Editors. The main publication will include the primary and secondary efficacy outcomes, safety outcomes, inflammation data, and microbiome data. Subsequent publications may be considered by the study investigators at the conclusion of the trial.

## Discussion

This manuscript describes the design and rationale for the PROTECT trial. The short-term objectives of the trial are to (1) assess the impact of prophylactic ceftriaxone on the incidence of EOP occurring < 4 days after initiation of mechanical ventilation in comatose survivors of OHCA, (2) quantify the ceftriaxone’s effect on T cell-mediated inflammation, (3) determine if prophylactic ceftriaxone alters the innate microbiome and resistome, and (4) collect preliminary data needed to design a definitive trial powered for functional outcome or mortality. The long-term objective is to conduct a large, multicenter trial examining functional outcome and mortality.

The ANTHARTIC trial reported a 47% reduction in EOP when intravenous amoxicillin-clavulanate was administered prophylactically for 2 days following OHCA.^18^ Our study will extend ANTHARTIC’s findings in several critical ways: (1) inclusion of OHCA survivors with all initial heart rhythms, (2) use of a lower-risk antibiotic available in the USA that has not previously been tested after OHCA, (3) study of the anti-inflammatory effects of ceftriaxone to determine a mechanism by which it improves clinical outcomes, and (4) complete metagenomic assessment of bacterial resistomes pre- and post-ceftriaxone prophylaxis.

In a rat model, ceftriaxone reduced IFN-γ and TNF-α in the injured parietal cortex and improved learning and spatial memory function [34]. Ceftriaxone also reduced IFN-γ and IL-17 secretion in a mouse model of multiple sclerosis by altering antigen presentation and activation of myelin-specific T lymphocytes [35]. Other studies have shown ceftriaxone dampens excitotoxicity by decreasing glutamatergic activity [34, 36–39]. Excitotoxicity may be a mechanism of injury following OHCA, so ceftriaxone’s ability to attenuate this response is promising [40].

T cells promote neuroinflammation and neuronal cell death via IFN-γ and TNF-α [40–43]. We found incubation of human CD3+ T cells with ceftriaxone for 24 h increased expression of CD73, but not the number of cells expressing CD73 (Fig. 1). However, cell surface expression of CD73 (non-permeabilized cells) was increased on T cells treated with ceftriaxone as defined by shift towards higher immunofluorescence. Expression of CD73 was increased at the cell surface of both CD8 and CD4 positive CD3 T lymphocytes. CD73 is an adenosine-generating enzyme, and adenosine has potent immunosuppressive and anti-inflammatory effects in T cells. Consistent with this concept, we found that the level of CD73 inversely correlated with IFN-γ levels in both CD4+ and CD8+ T cells.

The effects of prophylactic antibiotics on bacterial resistance after OHCA are not known, but antibiotic resistance is a global concern [44]. We will assess emergence of resistant bacteria in both treatment and control patients. Control patients may be exposed to longer courses of broad-spectrum antibiotics due to higher rates of pneumonia. We will create data on drug concentrations in stool, before-and-after effects of ceftriaxone on expression of resistance genes, and on richness, diversity, and relative abundance of taxa.

Earlier studies in acutely brain-injured patients found antibiotic prophylaxis did not induce bacterial resistance using limited analyses [15–17]. The ANTHARTIC trial evaluated intestinal acquisition of multidrug-resistant bacteria on day 7 on solid selective media using stool samples, limiting their observations to conditionally cultivatable organisms [18]. No difference was found following 2 days of amoxicillin-clavulanate administration compared to placebo. The gold standard for evaluating bacterial resistance is resistome analysis with high-throughput sequencing, which will be utilized in our trial.

## Conclusions

The PROTECT trial is a phase II trial of prophylactic ceftriaxone for 3 days in comatose OHCA survivors. The trial will provide preliminary data needed for a definitive phase III trial, as well as novel data on ceftriaxone’s anti-inflammatory effects and its impact on the microbiome. Our ultimate goal is to improve survival and quality of life in survivors of OHCA.

### Trial status

This manuscript describes the PROTECT trial protocol version #3 dated 6/11/21. Recruitment will begin in August 2020 and the anticipated completion is August 2023.

## Abbreviations

ANTHARTIC: Antibiotherapy during Therapeutic Hypothermia to Prevent Infectious Complications
CPC: Cerebral performance category
CPR: Cardiopulmonary resuscitation
EOP: Early-onset pneumonia
DSMB: Data Safety Monitoring Board
DSMP: Data Safety Monitoring Plan
EFIC: Exception from Informed Consent
INTCAR: International Cardiac Arrest Registry
IRB: Institutional Review Board
LAR: Legally authorized representative
mRS: Modified Rankin scale
OHCA: Out-of-hospital cardiac arrest
PROTECT: Ceftriaxone to PRevent pneumOnia and inflammaTion aftEr Cardiac arresT
ROSC: Return of spontaneous circulation
TTM: Targeted temperature management
